# Double blind, randomized controlled trial, to evaluate the effectiveness of a controlled nitric oxide releasing patch versus meglumine antimoniate in the treatment of cutaneous leishmaniasis [NCT00317629]

**DOI:** 10.1186/1745-6215-7-14

**Published:** 2006-05-15

**Authors:** Sandra Y Silva, Ligia C Rueda, Marcos López, Iván D Vélez, Christian F Rueda-Clausen, Daniel J Smith, Gerardo Muñoz, Hernando Mosquera, Federico A Silva, Adriana Buitrago, Holger Díaz, Patricio López-Jaramillo

**Affiliations:** 1 VILANO Group. Research Institute, Fundación Cardiovascular de Colombia (FCV), Floridablanca, Santander, Colombia; 2 Department of Chemistry, University of Akron, Akron, Ohio, USA; 3 Program for the Study and Control of Tropical Diseases, PECET, Universidad de Antioquia, Medellín, Antioquia, Colombia; 4 Universidad Industrial de Santander, Bucaramanga, Santander, Colombia; 5 Secretaría de Salud de Santander, Colombia; 6 Facultad de Medicina, Universidad de Santander, Bucaramanga, Santander, Colombia

## Abstract

**Background:**

Cutaneous Leishmaniasis is a worldwide disease, endemic in 88 countries, that has shown an increasing incidence over the last two decades. So far, pentavalent antimony compounds have been considered the treatment of choice, with a percentage of cure of about 85%. However, the high efficacy of these drugs is counteracted by their many disadvantages and adverse events. Previous studies have shown nitric oxide to be a potential alternative treatment when administered topically with no serious adverse events. However, due to the unstable nitric oxide release, the topical donors needed to be applied frequently, making the adherence to the treatment difficult. The electrospinning technique has allowed the production of a multilayer transdermal patch that produces a continuous and stable nitric oxide release. The main objective of this study is to evaluate this novel nitric oxide topical donor for the treatment of cutaneous leishmaniasis.

**Methods and design:**

A double-blind, randomized, double-masked, placebo-controlled clinical trial, including 620 patients from endemic areas for Leishmaniasis in Colombia was designed to investigate whether this patch is as effective as meglumine antimoniate for the treatment of cutaneous leishmaniasis but with less adverse events. Subjects with ulcers characteristic of cutaneous leishmaniasis will be medically evaluated and laboratory tests and parasitological confirmation performed. After checking the inclusion/exclusion criteria, the patients will be randomly assigned to one of two groups. During 20 days Group 1 will receive simultaneously meglumine antimoniate and placebo of nitric oxide patches while Group 2 will receive placebo of meglumine antimoniate and active nitric oxide patches. During the treatment visits, the medications will be daily administered and the presence of adverse events assessed. During the follow-up, the research group will visit the patients at days 21, 45, 90 and 180. The healing process of the ulcer, the health of the participants, recidivisms and/or reinfection will also be assessed. The evolution of the ulcers will be photographically registered. In case that the effectiveness of the patches is demonstrated, a novel and safe therapeutic alternative for one of the most important public health problems in many countries will be available to patients.

## Background

Cutaneous Leishmaniasis (CL) is a worldwide disease that is endemic in 88 countries [1]. It is estimated that 1.5 million people suffer from CL annually and that more than 350 million are at risk of contracting the infection [2-4]. In America, 60,000 new cases of CL are reported annually [5], being endemic in 20 of its 22 countries and in 2 islands of the Caribbean [2]. Currently, CL has affected more than 500 U.S. Army soldiers serving in Iraq [6]. In the Andean region, the incidence of Leishmaniasis has been increasing dramatically over the last two decades; reaching more than 14,000 cases per year from 1996–98 [7]. In Colombia 6,500 cases have been reported [8]. The increase in the reported cases of CL in Colombia has been related to factors such as migration, deforestation, the multiplication of illicit plantations, the armed political conflict and the behavioral changes of the vector. The main strains of *Leishmania *in Colombia are *L. panamensis*, *L. brazilensis*, *L. infantum *and *L. guyanensis*, which are distributed throughout the entire national territory, predominantly in the rural areas [9]. CL is caused by intracellular protozoan parasites of the genus *Leishmania *[1] and is transmitted to humans through the bite of a small percentage of the species of phlebotomus and lutzomyia sandflies classified to date [9]. In the digestive system of the sandflies, this dimorphic parasite presents an extracellular flagellated form called a promastigote, which upon its release in the host blood, is phagocyted by the macrophage, losing its flagella and turning into an amastigote [10]. Dogs, rodents and didelphidae are the natural hosts of the parasite while man is an incidental host [11]. This zoonosis has suffered an interesting urbanization phenomenon, changing from an eminent rural entity affecting mainly men of an active age, to a disease that is affecting all people, especially children [8,12]. The characteristic lesions of this disease are ulcers that heal spontaneously over a period of three months to a year, depending on the isolate, and that leave a flat, atrophic and depigmented scar [13-15]. The CL, especially the one produced by *L. brazilensis *can evolve into mucocutaneous Leishmaniasis (MCL), which has a worse prognosis owing to the deforming character of its lesions [16]. The spontaneous cure of these lesions allows for the acquisition of partial resistance to reinfection, which could explain the higher pathogenicity observed in the children and young adult population [12]. Previous studies have shown a higher incidence of CL and a poor response to treatment in the children population [17]. The program of epidemiological vigilance in Colombia requires that the probable cases of CL (identified by ulcer features and by the patient's origin) be confirmed by microscopic direct examination of a secession sample obtained from the ulcer, if these are negative, by biopsy of the wound. Once confirmed, the cases must be notified to the Local Health Secretary using clinical-epidemiological records. This institution, in charge of the epidemiological vigilance, studies the sources of transmission and distributes the medication to the people affected. Currently, various aspects are considered when treating CL, among which, the risk of developing MCL, the grade, localization, number, size, evolution and persistence of the lesions, are the most important [18]. For more than 60 years, the pentavalent antimony compounds: sodium stibogluconate, (Pentostan^®^, produced by Glaxo-Wellcome) and meglumine antimoniate, (Glucantime^®^, produced by Sanofi-Aventis) have been considered the treatments of choice for this disease [19]. Studies made in Colombia reported a percentage of cure of 85%, using meglumine antimoniate [20,21]. Despite the efficacy of these drugs is high, they present many disadvantages such as parenteral administration, and, reversible secondary effects such as nausea, vomiting, muscular and abdominal pain, cardiac problems, a rise in the concentration of hepatic aminotransferases, and chemical pancreatitis [22,23]. Additionally, the adherence to the treatment is affected by its duration (several weeks) and its availability by the restriction in its distribution. Therapeutic alternatives of second line have been proposed; amphotericin B and pentamidine have been used with excellent results, nevertheless their high cost, little availability, the necessity to hospitalize the patients for their administration and the severity of their secondary effects have limited their use [23,24]. In the last decade new treatments for CL have been developed, using oral agents such as mefloquine, itraconazole, miltefosine, paromomycin, ketoconazole, allopurinol and dapsone, however, they have not shown enough evidence of their effectiveness [19,21,25,26]. In an effort to develop a topical treatment for CL, paromomycin has been used in different preparations. However, healing rates achieved with this medication have not been higher than conventional treatments, even when compared with placebo [27,28]. In several studies, *in vitro *and *in vivo*, it has been demonstrated that nitric oxide (NO) is effective to eliminate various strains of *Leishmania *in its amastigote form [29-35]. The production of NO from the oxidation of L-arginine caused by the inducible nitric oxide synthase (iNOS) constitutes one of the most important defense mechanisms of the macrophages [36], in which two oxidative forms of defense against *Leishmania *have been identified. During the first phase of infection, in response to the phagocytosis process, some promastigotes are eliminated due to the release of the superoxide ion, a process which is catalyzed by the NADPH oxidase [29]. Those promastigotes that survive this defense mechanism evolve into amastigotes, activating the production of IL-12 in the macrophages and promoting the presentation of the antigens of *Leishmania *[29] to the T helpers 1 lymphocytes that enhance the cytotoxic activities of the macrophages toward the intracellular parasites via the interferon gamma (INFγ) and the tumor necrosis factor alpha (TNFα) by promoting the production of NO catalyzed by iNOS [31-33]. A recent study shows a higher activity of iNOS in the macrophages of subjects infected with CL, suggesting a vital role of NO in the immunological activity against *Leishmania *[34]. In Studies with rodents resistant to *Leishmania *infection (C57BL/6), where *L. major, L. chagasi *or *L. donovani *were inoculated, the application of iNOS inhibitors like N^G^-monomethyl-L-arginine (L-NMMA) caused a higher rate of survival and virulence of the parasites in macrophages [33,35,37,38]. After inoculating *L. major *in mice with the genetic susceptibility to develop infections with *Leishmania *(BALB/C), no activity of iNOS was observed. However, the application of IL-12, was able to control the infection by activating iNOS [31]. In humans, several clinical trials have been realized with topical treatments containing NO donors [39,40]. In Ecuador, our group developed and tested a NO generating topical cream with S-nitroso-N-acetylpenicillamine (SNAP), evidencing a beneficial effect in the management of this type of ulcers with no reports of any serious adverse event.

Nevertheless, due to the unstable nitric oxide release, the cream had to be applied frequently (4 times a day) making the adherence to the treatment difficult [39]. In Syria, another group used potassium nitrate acidified with salicylic acid and ascorbic acid for the topical treatment of *L. tropica *[40]. *In vitro*, this NO generating mixture destroyed the amastigotes and promastigotes of *Leishmania*; however, *in vivo*, the study of 40 patients presented inconsistent results, reducing the size of the ulcer in 28% of the subjects and healing only 12%. The discrepancy in these results is believed to be due to the technique used to obtain the NO. The acidification of nitrite produces an instant blast of NO, but its release is not maintained over a long period of time[40]. The difficulty of controlling the liberation of NO has created the necessity of looking for new techniques to regulate its release. The nanofiber polymers produced by the electrospinning technique have been studied in order to guarantee the constant release of pharmaceuticals on the lesion. In the electrospinning process, a high voltage is used to create an electrically charged jet of polymer solution, which dries and solidifies to leave behind a dry polymer fiber [41]. As this jet travels through the air, the solvent evaporates leaving behind a charged fiber that can be electrically deflected and collected on a metal screen [42,43]. Fibers with a variety of cross sectional shapes and sizes are produced from different polymers. With this technique, the encapsulation or entrapment of several pharmaceuticals, enzymes and proteins has been successful. In a previous study, nanofiber patches were successfully used as releasing vehicles of tetracycline hydrochloride. The release of tetracycline was constant for a period of 5 days [41,43]. Using the same model, a multilayer transdermal patch has been produced, in which nitrite is bound to an ion exchange resin (DOWEX) and electrospun into a polyurethane nanofibers layer. A solution containing Waterlock^® ^superabsorbent and polyurethane is electrospun on top of the nitrite-DOWEX layer. The ascorbic acid entrapped in the polyurethane solution is electrospun onto a third layer, with another layer of Waterlock^® ^superabsorbent and polyurethane as the fourth and final one. Upon hydration, this Nitric Oxide Releasing Patch (NOP) produces a stable release of 3.5 μmol of NO during 12 hrs [41,44,45]. In a pilot study, developed in Landazuri, Santander, Colombia, a placebo-controlled clinical trial was conducted with 35 patients who presented 68 ulcers produced by *L. panamensis*. Using the NOP, a 65% improvement was observed in the treated ulcers, with only a 25% improvement in the placebo group (p = 0.001). In this pilot study the unique adverse event described was pruritus in the area where the patch was applied (unpublished data). Taking into account the wide distribution of CL, the changes in its form of transmission and the difficulty related with the availability of medication, this study proposes to investigate whether the NO donor transdermal patch, produced by electrospinning is, at least, as effective as the meglumine antimoniate for the treatment of CL, with less adverse events and a lower cost, constituting therefore an effective therapeutic alternative. In case that the effectiveness of the NOP is demonstrated in this study, a novel and safe therapeutic alternative of easy access and higher adherence for one of the most important public health problems in our country will be made available.

### Objectives

#### General objective

To evaluate the effectiveness and safety of NOP in the treatment of CL compared with meglumine antimoniate (Glucantime^®^).

#### Specific objectives

1. To evaluate the healing rate of CL ulcers using a NOP compared with the plan of treatment with meglumine antimoniate recommended by the health ministry.

2. To identify adverse events associated with the application of NOP and compare them with the ones produced by the treatment with meglumine antimoniate.

3. To identify and compare the recidivisms that may occur with both NOP and meglumine antimoniate.

4. To advance in the search of a therapeutic alternative for CL in Colombia.

### Design

Double blind, randomized, double-masked, placebo-controlled clinical trial, comparing nitric oxide releasing patches with meglumine antimoniate.

### Sample size

The sample was calculated according to the arccosine formula using a power of 80% and a type 1 error of 0.05%. Assigning a successful rate of 85% for meglumine antimoniate and 75% for NOP, 558 patients will be needed. After adjustment for a loss rate of 5%, the total of patients that must be recruited is 620 (310 patients per treatment group).

### Population

The population will be composed from two regions of Colombia. The first one is an endemic zone in Santander, Colombia, located between the Magdalena Valley and the East Mountain Range, which includes the municipalities of Landazuri, El Carmen, San Vicente, El Playon and Rionegro. The second region is an endemic zone in North Tolima, which includes the municipalities of Chaparral, San Antonio, Libano, Falan, Palocabildo and Mariquita.

### Selection of the patients

#### Inclusion criteria

1. Men and women between 18 and 50 years old

2. Cutaneous ulcers of more than two weeks of evolution

3. Positive parasitological diagnosis for CL

4. Patients that voluntarily accept to participate in the study and sign the informed consent.

5. Disposition to attend all the visits punctually (initial, treatment and follow-up)

6. Acceptation of not using any other treatment for CL while in the study

#### Exclusion criteria

1. Pregnant women

2. Presence of any condition or disease that compromises the patient immunologically (ie. diabetes, cancer, etc.) or, any other, that, based on the judgment of the researcher, could alter the course of CL.

3. Diffuse CL or more than five active lesions.

4. Mucocutaneous leishmaniasis (no lesion must be located less than 2 cm from the nasal, uro-genital, and/or anal mucous membranes or from the edge of the lips).

5. Visceral leishmaniasis.

6. Complete or incomplete treatment with antimony compounds in the last three months.

7. Patients with history of hepatic, renal or cardiovascular disease.

8. Mentally or neurologically disabled patients that are considered not fit to approve their participation in the study.

### Study development

#### Logistic phase

This phase will last 4 months and will include the following activities:

1. Training of the personnel that will participate in the study.

2. Acquisition of the materials required for the development of the project.

3. Elaboration of flyers, promotional and educative material, procedures manual and case report forms (CRFs).

4. Treatment randomization.

The treatment randomization will be realized by the epidemiologist of the Cardiovascular Foundation of Colombia. This randomization will be done in blocks in order to keep the size of the treatment groups similar, to avoid long sequences of the same treatment and to balance when possible some of the bias inherent to the simple randomization process. Additionally, this randomization in blocks will facilitate the execution of interim analyses.

#### Recruitment phase

This phase will take 22 months. In the selected municipalities, the health personnel that work in hospitals and health centers will receive training regarding the disease and the study methodology. Simultaneously an epidemiological focus study will be done with the leaders of the community and the health personnel to identify the geographic and demographic conditions with the purpose of developing strategies for the recruitment of possible cases of leishmaniasis. Subsequently, the subjects that present active ulcers with more than 2 weeks of evolution, with or without parasitological confirmation of CL, will be invited by the health promoters to attend the screening visit (Table 1).

**Table 1:** Schedule of activities

**PROTOCOL ACTIVITIES**	**Screening Visit**	**Initial Visit**	**Treatment visits**	**Follow-up visits**			
				**Visit 1**	**Visit 2**	**Visit 3**	**Visit 4**
**SCHEDULE OF VISITS**		0	1–20	21+3	45 ± 7	90 ± 14	180 ± 14
Clinical History	X	X		X	X	X	X
Physical Examination	X	X		X	X	X	X
Inclusion/Exclusion criteria	X						
Informed Consent	X						
Direct test	X						
Sampling of cultures		X					
Laboratory tests		X		X			
Randomization		X					
Treatment administration		X	X				
Clinical assessment of the lesion	X	X	X	X	X	X	X
Measurement of ulcers		X		X	X	X	X
Photographic registration		X		X	X	X	X
Education of patients	X	X	X	X	X	X	X
Handing over of diary		X					
Evaluation of effectiveness				X	X	X	X
Documentation of adverse events	X	X	X	X	X	X	X
Recording of concomitant medications	X	X	X	X	X	X	X

### Screening visit

During this visit a complete clinical history will be elaborated and data about antecedents of leishmaniasis obtained (administered treatment, localization, number of lesions, etc). A full medical evaluation will be realized based on universally accepted techniques. The inclusion/exclusion criteria will be applied and the selected candidates informed about the study after which they will sign an informed consent. For those subjects with ulcers of more than two weeks of evolution without parasitological diagnosis a direct test will be performed by the parasitologist. If the first direct test is negative, it will be repeated up to three times; after which, in case of negative results, a biopsy will be performed. In case leishmaniasis is not confirmed the patient will be remitted to the original health center.

### Initial visit

The included patients will be randomly assigned in one of two groups. A culture in Novy-Nicolle-McNeal (NNN) medium will be taken for strain identification and a blood sample withdrawn from the antecubital vein for hepatic enzymes, creatinine, and pancreatic amylase determination. During this visit, a complete medical evaluation will be performed, the ulcer will be measured and a picture will be taken. Before taking the picture, a graduated rule will be placed next to the ulcer along with a sticker marked with the identification code of the participant, and the date of the visit. The first NOP (active or placebo) will be applied covering the lesion and the first shot of meglumine antimoniate (active or placebo) administered. The patients will receive information on how not to damage the patches and how to identify and report adverse events. To help the patients to do so, they will receive a diary to register them. The two groups randomly composed will be divided as follows:

Group 1: During 20 days this group will receive simultaneously intramuscular (IM) meglumine antimoniate (Glucantime^® ^20 mg/kg/day with a maximum dose of 3 ampoules per day); and an NOP placebo.

Group 2: During 20 days this group will receive simultaneously placebo of IM meglumine antimoniate (5–15 cc/day) and an active NOP.

### Treatment visits

The patients will visit the health center daily during 20 days to receive both the NOP (placebo or active) and meglumine antimoniate (placebo or active). Daily, the subjects and their adherence to the treatment will be assessed and the data collected. If any adverse event is detected, the patient will be referred immediately to the medical staff that will make an evaluation and report it to the adverse event committee that will take the final decision in each case.

### Follow-up visits

This phase will last 10 months. During the follow-up, the patients will be seen by the research group in 4 opportunities. The first visit will take place the day after the end of the treatment (day 21) in which new blood samples will be taken for biochemical determinations. The second, third and fourth visits will be realized on day 45, 90 and 180 respectively. During these visits the healing process of the ulcer, the presence of recidivisms and/or reinfection and the health of the participants will be assessed. The evolution of the ulcers will be photographically registered.

The maximum and minimum diameters of the ulcer will be measured using a graduated ruler, and the induration using the ballpoint pen technique. The ulcer and induration areas will be calculated separately, and then registered in the CRF. The evaluation of the clinical response will be based on the ulcer showing the least improvement.

1. Complete clinical response: Complete reepithelization of the ulcer and disappearance of the induration.

2. Clinical Improvement: Reduction of more than 50% of the ulcer and the induration areas in relation to the last clinical evaluation.

3. Absence of clinical response: Increase or reduction of less than 50% of the ulcer and the induration areas in relation to the last clinical evaluation.

4. Therapeutic failure:

a. Increase in the size of the ulcer by more than 50% in relation to the last clinical evaluation

b. Presence of the ulcer three months after the beginning of the treatment.

c. Reactivation: Appearance of a lesion on the edge or in the center of the scar, with positive parasitological diagnosis, after a period of complete reepithelization.

d. Affection of the mucous membranes: Presence of affections of the mucous membranes during the treatment, at the end of it or in the follow-up visits.

5. Reinfection: Activation of an ulcer in an area different from the original lesion.

For subjects whose treatment will have been considered a failure the code will be broken and they will be remitted to their original health center to look for other therapeutic approach.

### Procedures

#### Physical examination

A complete physical examination will be realized and vital signs will be measured.

### Blood samples withdrawn

Blood samples will be withdrawn from the antecubital vein to perform the following biochemical analyses:

1. Creatinine by spectrophotometry

2. Alanine transaminase (ALT) by spectrophotometry

3. Aspartate transaminase (AST) by spectrophotometry

4. Pancreatic amylase by spectrophotometry

### Sampling technique for the direct test of CL

A direct test is performed to confirm the presence of cells with amastigotes in a dermis scrape [46,47]; the sample must be, in so far as it is possible, free of blood, cellular detritus or pus. If the patients present several lesions, the sample must be taken from the one with the shortest time of evolution, the biggest induration area and/or the least purulent discharge. If the lesion has a scab it must be removed to improve the quality of the sample. The sample can be taken from the active edge or from the bottom of the ulcer. When the sample is taken from the active edge asepsis must be realized with alcohol at 70% and hemostasis then performed using the first and second fingers to make sure that there is no blood in the sample. A small incision of about 3 mm in length and depth is made with a scalpel in the active edge parallel to the edge of the ulcer. With the sharp side of the scalpel, a scrape is done in the dermic wall of the incision to obtain tissue. The extracted material is extended on two microscope slides. The sample is dried at room temperature, and then fixed with methanol and stained with Giemsa, Wright or Field. Using immersion oil the sample is observed under a microscope with a 100× lens, the presence of amastigotes of *Leishmania *assessed and their structure verified (nucleus, kinetoplast and cell membrane). If the sample is taken from the center of the lesion the same hemostatic technique must be used, the scab removed, and the bottom of the ulcer well cleaned using the sharp side of the scalpel to prevent the presence of cellular detritus and/or purulent material. This procedure must be continued until the granulomas are seen at the bottom. With the same technique used to process the samples from the active edge the presence of amastigotes of *Leishmania *is verified.

### Technique for the sampling of cultures

The sample for the culture may be obtained by suctioning the ulcer active edge or by extracting a fragment of tissue which is then macerated in a phosphate-buffered saline solution (PBS) with antibiotics (1000 UI of crystalline penicillin per cc), before it is put in the culture medium. A tuberculin syringe with a thin needle (26G), containing 0.3 cc of PBS solution with antibiotics is used in the suction technique. Previous asepsis of the ulcer with alcohol at 70%, a needle is introduced into the dermis and through rotating movements a small amount of tissue is macerated by the needle bevel during about a minute, after which it is suctioned into the syringe. The sample is deposited in aseptic conditions into a NNN culture medium and incubated at 26°C during 4 weeks [48,49]. Every week a drop is extracted from the culture medium and placed between two slides to be observed under a microscope. In case that promastigotes are not found, the cultures are rejected as negative [46]. The strains are identified by species using the monoclonal antibodies developed by Dr. Diane Mc Mahon Pratt [50, 51]

### Evaluation and management of adverse events

During the treatment and the follow-up visits, the patients will be asked about adverse events. Each adverse event will be classified by the physician as serious or non-serious(Table 2 and Table 3). A serious adverse event should meet one or more of the following criteria:

**Table 2:** Toxicity grading scale for determining the severity of clinical adverse events

**SYSTEMIC**				
	**Grade 1 (Mild)**	**Grade 2 (Moderate)**	Grade 3* (Severe)	Grade 4* (Life-threatening)
Fever: oral^1^	38 – 38.5°C	38.6 – 39.5°C	39.6 – 40.5°C	>40.5°C
Headache^1^	Mild; no Rx required	Transient, moderate; Rx required	Severe; responds to initial narcotic Rx	Intractable; required repeated narcotic Rx
Allergic reaction^1^	Pruritus without rash	Localized urticaria	Generalized urticaria; angioedema	Anaphylaxis

**GASTROINTESTINAL**				

Nausea^1^	Mild discomfort; maintains reasonable intake	Moderate discomfort; intake decreased significantly; some activity limited	Severe discomfort; no significant intake; activities limited	Minimal fluid intake
Vomiting^2^	1 episode in 24 hours	2 – 5 episodes in 24 hours	> 6 episodes in 24 hours or needing IV fluids	Physiologic consequences requiring hospitalization or parenteral nutrition
Diarrhea^2^	Mild or transient; 3–4 loose stools/day or mild diarrhea lasting < 1 week	Moderate or persistent; 5–7 loose stools/day or diarrhea lasting > 1 week.	>7 loose stools/day or bloody diarrhea; or orthostatic hypotension or electrolyte imbalance or >2 LIV fluids required	Hypotensive shock or physiologic consequences requiring hospitalization
Oral discomfort^2^	Mild discomfort; no difficulty swallowing	Some limits on eating/drinking	Eating/talking very limited; unable to swallow solid food	Unable to drink fluids; requires IV fluids

**CARDIOVASCULAR**				

Cardiac rhythm^2^	----	Asymptomatic, transient signs, no Rx required	Recurrent/persistent symptomatic Rx required	Unstable dysrythmia; hospitalization and Rx required
Hypertension^1^	Transient increase > 20 mmHg; no Rx	Recurrent, chronic > 20 mmHg, Rx required	Requires acute Rx; no hospitalization	Requires hospitalization
Hypotension^1^	Transient orthostatic hypotension, no Rx	Symptoms corrected with oral fluids	Requires IV fluids; no hospitalization required	Requires hospitalization

**MUSCULOSKELETAL**				

Arthralgia^2^(joint pain)	Mild pain not interfering with function	Moderate pain, analgesics and/or pain interfering with function but not with ADL	Severe pain; pain and/or analgesics interfering with ADL	Disabling pain
Myalgia^2^	Myalgia with no limitation of activity	Muscle tenderness (at other than injection ^2^site) or with moderate impairment of activity	Severe muscle tenderness with marked impairment of activity	Frank myonecrosis

**RESPIRATORY**				

Cough^3^	Mild, relieved by non-prescription medication only	Symptomatic requiring narcotic prescription medication	Symptomatic and significantly interfering with sleep or ADL	NA
Dyspnea^3^	Dyspnea on exertion, but can walk one flight of stairs without stopping	Dyspnea on exertion, but unable to walk 1 flight of stairs or 1 city block (0.1 Km) without stopping	Dyspnea interfering with ADL	Dyspnea at rest requiring oxygen therapy; intubation/ventilator indicated

^1 ^= WHO Toxicity Grading Scale; ^2 ^= DMID, NIH, Adult Toxicity Table; ^3 ^= CTCAE v3.0; *Report Grade 3 and Grade 4 adverse events to the investigators in charge.

ADL: Activities of daily living

Ref: Toxicity Grading based on CTCAE v3.0 DCTD, NCI, NIH, DHHS December 12, 2003 .

**Table 3:** Toxicity grading scale for determining the severity of laboratory adverse events

**Parameter**	**Normal Range#**	**Grade for Abnormal Results (Value or Change from Reference)**			
	**(Grade 0)**	**Mild (Grade 1)**	**Moderate (Grade 2)**	Severe (Grade 3)*	Severe (Grade 4)*
**ALT (GPT)**	5 – 40 U/L	>ULN – 2.5 × ULN	>2.5 – 5.0 × ULN	>5.0 – 20.0 × ULN	>20.0 × ULN
**AST (GOT)**	6 – 35 U/L	>ULN – 2.5 × ULN	>2.5 – 5.0 × ULN	>5.0 – 20.0 × ULN	>20.0 × ULN
**Creatinine**	0.7 – 1.5 mg/L	>ULN – 1.5 × ULN	>1.5 – 3.0 × ULN	>3.0 – 6.0 × ULN	>6.0 × ULN
**Pancreatic Amylase**	<120 U/L	>ULN – 1.5 × ULN	>1.5 – 2.0 × ULN	>2.0 – 5.0 × ULN	>5.0 × ULN

# Normal lab ranges may vary from time to time depending on the reagent/kit used at local lab. Every time the normal ranges change, the laboratory will fax a copy of the "normal ranges" for that date to the Investigators. To respect confidentiality, the subject's name will be covered.

* Grade 3 or 4 lab value: Suspend treatment.

ULN: Upper limit of normal range

Ref: Toxicity Grading based on CTCAE v3.0 DCTD, NCI, NIH, DHHS December 12, 2003 .

1. Death

2. Life-threatening (i.e., immediate risk of death)

3. In-patient hospitalization or prolongation of existing hospitalization

4. Persistent or significant disability/incapacity

The presence of a serious adverse event that puts the patient's life at risk and/or requires immediate medical or surgical procedure will call for the discontinuation of the treatment and the initiation of the pertinent medical management of the patients. The investigator will notify the Adverse Event Committee (AEC) of the FCV of any serious adverse event within 24 hours of learning about it.

A non-serious adverse event will be classified as follows:

1. Mild: The patients are aware of their symptoms and/or signs, but those are tolerable. They do not require medical intervention or specific treatment.

2. Moderate: Patients present troubles that interfere with their daily activities. They require medical intervention or specific treatment.

3. Severe: The patients are unable to work or to attend their daily activities. They require medical intervention or specific treatment.

The possible relationship between the adverse events and the tested medication will be classified by the investigator on the basis of his/her clinical judgment and the following definitions:

1. Definitely related: Event can be fully explained by the administration of the tested medication.

2. Probably related: Event is most likely to be explained by the administration of the tested medication rather than other medications or by the patient's clinical state.

3. Possibly related: Event may be explained by the administration of the tested medication or other medications or by the patient's clinical state.

4. Not related: Event is most likely to be explained by the patient's clinical state or other medications, rather than the tested one.

All the events will be reported to the AEC that, depending on their criteria, will decide for the continuity or the withdrawal of the patient from the study and therefore the breaking of the code.

Although the project has been designed to minimize the inherent risks, any adverse event related to the study medications will be carefully evaluated by the AEC and the costs generated by the required treatment will be cover by the study.

### Data analysis phase

This phase will last 6 months. After completing all the data entry to the CFR, the results will be audited and the detected errors evaluated and corrected by the person in charge. The information will be entered in two different databases by two different people and the records will be compared to detect any discrepancy. The original CFR will be used to correct any mistake in the database.

For the statistical analysis, Stata 8.0 will be used. The descriptive analysis will be composed of medians and proportions according to the nature of the variables, with its respective 95% confidence intervals. As a dispersion measurement the standard deviation will be calculated. The distribution of the variables will be studied using the Shapiro-Wilk test. To detect any difference between the groups, a T-test or a Wilcoxon test will be performed according to its distribution. The categorical variables will be compared using the Chi2 test or the exact Fisher's test. If required, a model of multiple logistic regressions or a covariance analysis will be done.

Two interim analyses will be performed when 35% and 70% of the calculated sample is collected, with the objective of determining the differences in effectiveness and safety between the treatments.

### Endpoints

At the end of the study two endpoints will be evaluated:

1. Successful Treatment:

a. Complete reepithelization three months after the beginning of the treatment.

b. Absence of reactivation and affections of the mucous membranes during the 6 months of the study.

2. Treatment Failure:

a. Incomplete reepithelization three months after the beginning of the treatment.

b. Increase in the size of the ulcer by more than 50% in relation to the last clinical evaluation

c. Reactivation and/or affections of the mucous membranes during the 6 months of the study.

### Final report

At the end of the study the results will be evaluated and discussed and a final report presented to COLCIENCIAS, entity that is sponsoring the project. The relevant results will be published in both, national and international journals, and presented in congresses and scientific meetings.

### Ethical aspects

This study will be conducted in accordance with the Declaration of Helsinki and with the Colombian legislation as per the Resolution 8430/93 from the Ministry of Health. Prior to the admission of the patients in the study, the objectives and the methodology will be explained and the informed consent obtained. The study was approved by the Research Ethic Committee of the Cardiovascular Foundation of Colombia (Act# 105/January 28/2005). The right to confidentiality of the patients will be maintained in all the phases of the study.

## Competing interests

The author(s) declare that they have no competing interests.

## Authors' contributions

PLJ has made substantial contributions to the conception and design of the study, will be responsible for the overall administration and direction of the project, the analysis and interpretation of data and will give the final approval of the version to be published. DS, who participated in the design of the project, will be responsible for the overall administration and direction of the study, and the production and analysis of the NO releasing electrospun nanofiber patches. IDV has made substantial contributions to the conception and design of the study and to the drafting of the manuscript. He will also be responsible for analyzing and interpreting the final data.

ML was in charge of the development of the NOPs to determine their optimum dosage. He was also involved in the drafting of the manuscript and will participate in the analysis and interpretation of data. HM and HD were involved in the drafting of the manuscript and the planning of the clinical trial. He will also be responsible for overseeing its logistics and will interpret the final data. GM will be responsible for the parasitological analyses, biopsies, and the processing of the patients' samples. He will also participate in the acquisition, analysis and interpretation of data. FS-S, contributed to the conception and design of the study, performed sample size calculation and randomization and will analyze the data obtained from the clinical trial. SYS made substantial contributions to the conception and design of the study and was involved in drafting the manuscript. LCR contributed to the design of the study, was involved in drafting the manuscript, will be responsible for the recruitment and follow-up of the patients enrolled, and will also participate in the analysis and interpretation of data. CFR-C contributed to the design of the study, was involved in drafting the manuscript, will be responsible for the recruitment and follow-up of the patients enrolled, and will also participate in the analysis and interpretation of data. AB participated in the design of the study, will contribute to the acquisition of data, and will be responsible for overseeing the application of the protocol by monitoring the clinical trial to ensure compliance with the cGCP (current Good Clinical Practices). All authors read and approved the final manuscript.
